# Antimicrobial Efficacy of Morinda citrifolia, Nisin, and 2% Chlorhexidine Against Enterococcus faecalis: An In-Vitro Study

**DOI:** 10.7759/cureus.23206

**Published:** 2022-03-16

**Authors:** Sravani Nirmala, Surender L.R, Narender Reddy, Sainath D Reddy, Rakesh Reddy Chukka, Naresh Kumar K

**Affiliations:** 1 Conservative Dentistry and Endodontics, SVS Institute of Dental Sciences, Mahabubnagar, IND

**Keywords:** sodium hypochlorite, lantibiotic, microbiota, incubation, ethylene diamine tetraacetic acid, enterococcus faecalis, 2% chlorhexidine, colony forming units, nisin

## Abstract

Aim

The aim of this study was to evaluate and compare the antimicrobial activity of 2% chlorhexidine gluconate (2% CHX), *Morinda citrifolia* (*M. citrifolia*), and nisin (NI) all in gel forms against *Enterococcus faecalis* (*E. faecalis*)-infected root canals.

Methodology

Forty single-rooted mandibular premolars extracted for orthodontic reasons were decoronated and chemomechanical preparation of the root canal was performed. After sterilization, the samples were inoculated with *E. faecalis* for one week and grouped according to the medicament used namely, saline as the control group (Group-A), 2% CHX (Group-B), *M. citrifolia* (Group-C), and NI (Group-D). After 7days of incubation, in order to evaluate the effectiveness of the intracanal medicaments on the canal wall and its radicular dentin, the specimens dentin chips were retrieved and inoculated on brain heart infusion (BHI) blood agar plates from each tube and incubated at 37^°^C for 24 hours to obtain bacterial colony forming unit (CFU) count. The data was statistically analyzed using one-way ANOVA test and multiple comparisons among different groups were complemented by post hoc Tukey test.

Results

The CFU count indicating the number of viable bacterial colonies was found to be highest in Group-A (saline). Group-B (CHX 2%) showed the least CFUs followed by Group-D (NI) and Group-C (*M. citrifolia*).

Conclusion

In an attempt to overcome the disadvantages and toxic effects of a few commercially available intracanal medicaments and irrigants, the present study was aimed at using herbal extracts to evaluate and compare their antimicrobial efficacy with the commercially available medicaments against *E. faecalis*. Nisin was an effective antimicrobial agent and its action was found to be comparable with CHX.

## Introduction

Bacteria and their products play a crucial role in the initiation and sustaining of pulpal and periapical diseases. Chemomechanical preparation is one of the most important aspects of endodontic treatment in reducing the microflora and enhancing periapical tissue repair [1]. The primary pathogen isolated from root canal retreatment cases is *Enterococcus faecalis*. It has the ability to survive in the extreme environment [2-5]. It is resistant to antibacterial agents as it can penetrate to a depth of 800 µm [6,7]. It is one of the most resistant microorganisms against antimicrobial irrigants and intracanal medicaments and has been chiefly associated with persistent periapical infections in necrotic pulps and endodontically treated teeth [8,9].

Chlorhexidine gluconate (CHX) is known for its antibacterial activity in dentistry. In endodontics, it is effective both as irrigant and medicament. It inhibits Gram-positive and Gram-negative microorganisms [10]. Nisin (NI), an antimicrobial therapeutic peptide, is effective on microorganisms that show resistance to common antibiotics. NI, a type-A lantibiotic, has good antibacterial activity and is effective against *E. faecalis* [11]. *Morinda citrifolia* (*M. citrifolia*), commonly known as noni, is used as folk medicine and health drink. The juice has a broad range of therapeutic effects, including antibacterial, antifungal, antiviral, antitumor, antihelminthic, analgesic, hypotensive, anti-inflammatory, and immune-enhancing properties. Murray et al. concluded that 6% *M. citrifolia* can be used as an endodontic irrigant [12]. Intracanal medicament had been used as an interim appointment medicament and most commonly used were commercial synthetic medicaments. However, a greater part had been reported for its toxic effect, development of resistant strains, and exhaustion of immune response, and this has led to a paradigm shift from synthetic to naturally acquired medicaments. Herbal extracts have fewer side effects and are easily available and renewable in nature [13]. However, very few studies have examined the efficacy of NI and *M. citrifolia* as an intracanal medicament. Hence, the present study intends to evaluate the antibacterial efficacy of a chemical bisbiguanide CHX gluconate, an antibiotic peptide NI, and a natural herbal medicament *M. citrifolia* all in gel forms against *E. faecalis*.

## Materials and methods

Preparation of samples

Forty single-rooted mandibular premolars extracted for orthodontic reasons (Department of Oral and Maxillofacial Surgery, SVS Institute of Dental Sciences, Mahabubnagar) were stored in distilled water were decoronated at the cementoenamel junction (CEJ) using a diamond disc and the root length was standardized at 10 mm. The patency of the canal was checked with 10 K-file (Mani Inc, Yohara, Tochigi, Japan) and the working length was established 0.5 mm short of the apex. Canals were instrumented with a complete Pro Taper sequence till F3 (Dentsply, Maillefer, Switzerland) in crown-down technique with copious intracanal irrigation using 1 ml of 5.2% sodium hypochlorite (NaOCl) (Vishal Dentocare Pvt.Ltd, Ahmedabad, India), 1 ml of 17% EDTA (Ramesh Research Products, Kolkata, India), and 0.9% normal saline solution (Infutech Health Care Ltd, Indore, India) to remove the smear layer. The specimens were then cleaned in distilled water for 30 minutes in an ultrasonic bath dried, coated externally varnish, and sterilized in an autoclave for 30 minutes at a temperature of 121°C and 15 psi pressure.

Contamination of samples

The apex of each root was sealed using modeling wax and the root canal was inoculated with 5 µl of prepared bacterial suspension of *E. faecalis* strain ATCC 29212 in liquid broth till the canal orifice using a micropipette in a microbiological laminar flow safety cabinet (Department of Microbiology, SVS Institute of Medical Sciences, Mahabubnagar) and the coronal portion of each root was sealed with modeling wax, placed in sterile containers and were incubated at 37°C for about 21 days. On every 3rd day, the canals were reinoculated with fresh bacterial suspension in order to prevent medium saturation. Asepsis was maintained throughout the procedure. After an incubation of 21 days, the inoculated specimens were randomly divided into four groups (n=10) for the placement of medicament.

Antibacterial assessment

After 21 days of incubation, the canal contents were retrieved with the help of aseptic paper points and the samples were then randomly divided into four groups (n=10 each) for the placement of intracanal medicament. Group-A (Control), normal saline (0.9%). Group-B 2% chlorhexidine (Gluco-Chex 2% gel Cerkamed Poland) was used as an intracanal medicament in the present study. Group-C, *M. citrifolia* gel form (Noni powder, Jioo organics, Devpuri Meerut, Uttar Pradesh) was prepared by weighing, 6 g (6%) of powder which is dissolved in distilled water in a sterile container in order to obtain a concentration of 600 mg/ml. To this preparation hydroxyl ethyl cellulose (Akshar Chem Exim Co. Pvt. Ltd) was added to obtain a gel form. Group-D, NI gel form (J.J.D Enterprises, Ghaziabad, Uttar Pradesh) was prepared by weighing 2.5 g of powder which is dissolved in distilled water in a sterile container in order to obtain a concentration of 250 mg/ml. To this preparation, hydroxyl ethyl cellulose was added to obtain a gel form. 5 µl of prepared medicament was injected into root canals of all the samples and sterile modeling wax was used to seal the teeth and incubated at 37°C for 7 days.

After 7 days of incubation, in order to evaluate the degree of infection on the canal wall and its radicular dentin, the specimens dentin chips were retrieved from the entire root canal length, i.e., 10 mm of the standard root length using Gates-Glidden drills no. 4 followed by 5 which were then transferred into 1 ml of sterile Eppendorf tubes containing sterile distilled water. This procedure was repeated for all the samples. The dentinal chips were allowed to sediment for about 5 minutes, after which the supernatant was discarded and the 5 µl of the sediment was inoculated on brain heart infusion (BHI) blood agar plates from each tube and incubated at 37°C for 24 hours to obtain bacterial colony forming units (CFU) count, which was made by agar plate culture.

Statistical analysis

The data was statistically analyzed using one-way ANOVA test and multiple comparisons among different groups were analyzed by post hoc Tukey test. The level of significance was established at P<0.05.

## Results

**Figure 1:**
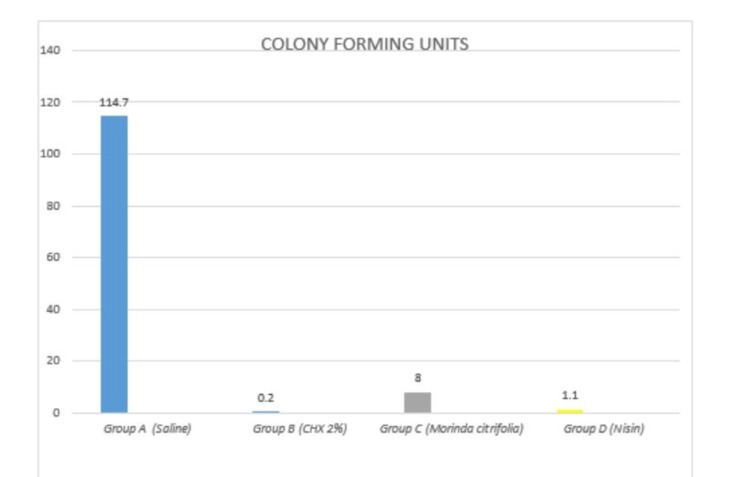
The simple median bar diagram depicting mean comparison between experimental groups CHX: chlorhexidine

**Figure 2:**
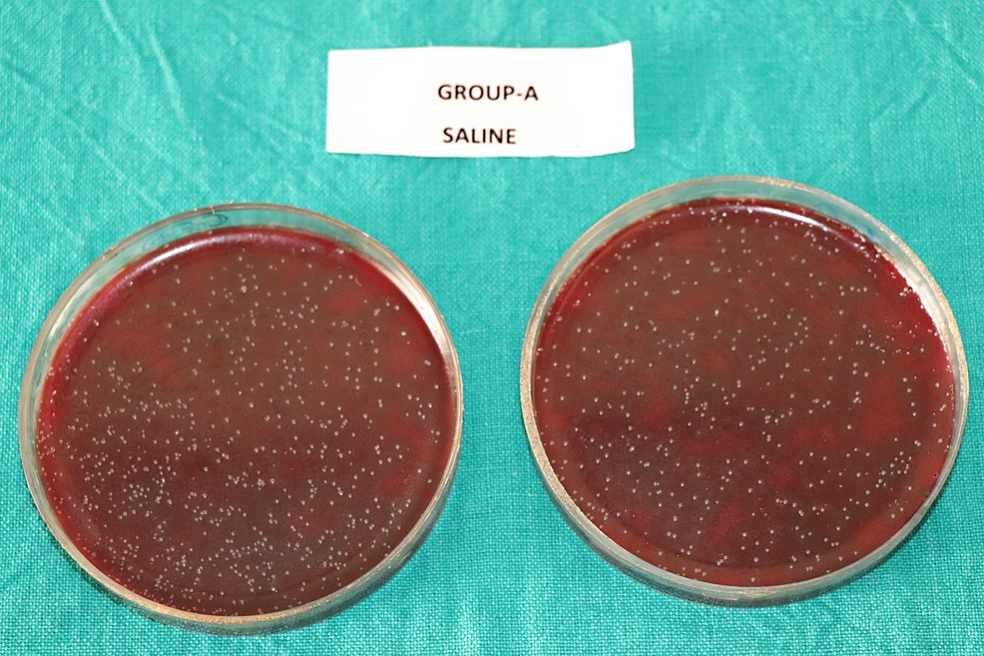
Colony forming units in Group-A (Saline)

**Figure 3:**
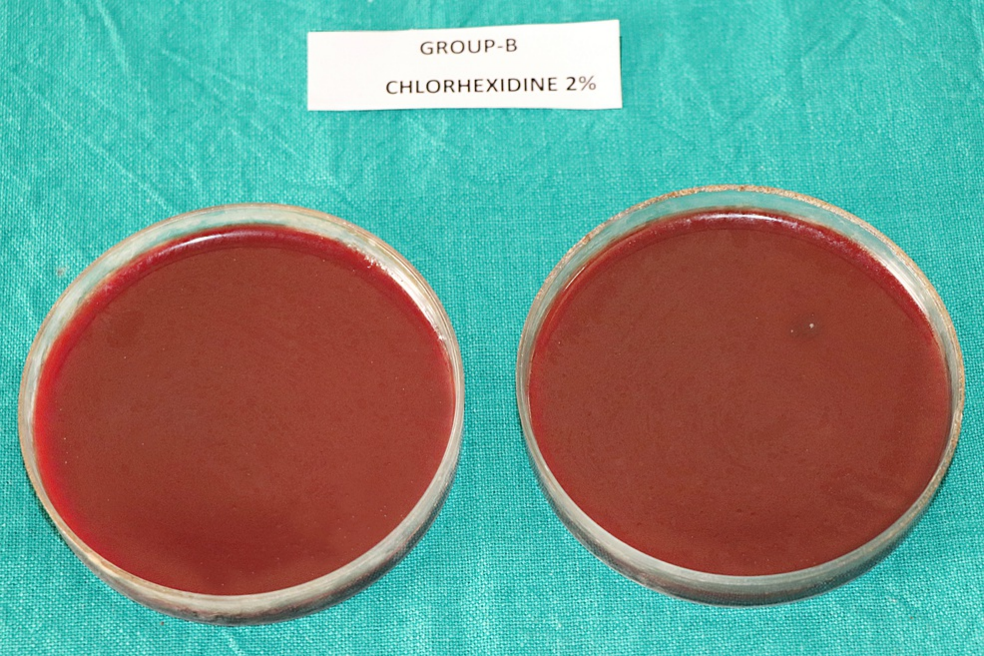
Colony forming units in Group-B (CHX 2%) CHX: chlorhexidine

**Figure 4:**
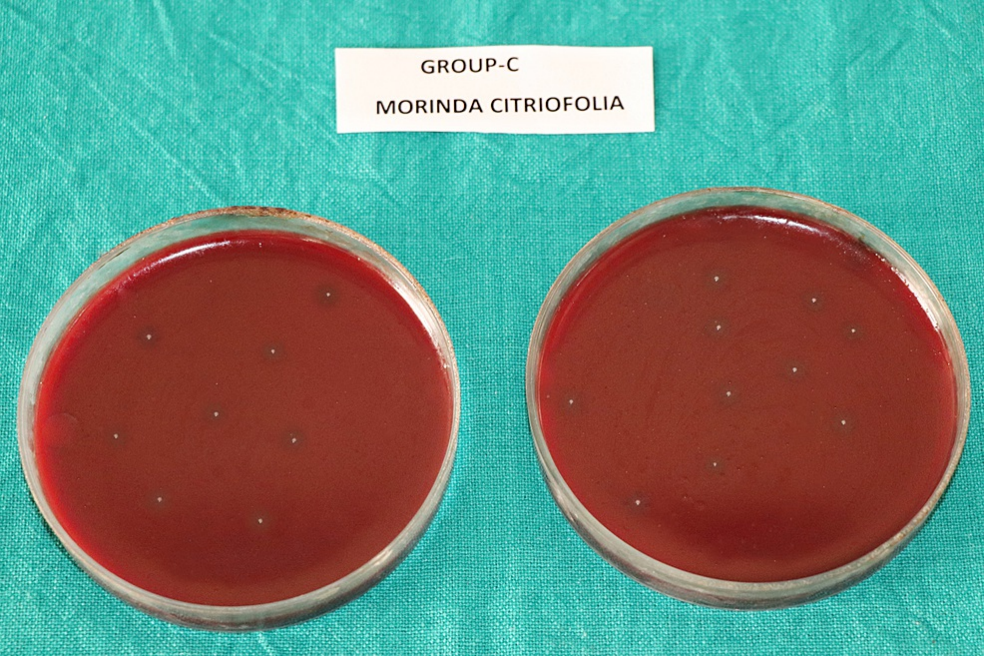
Colony forming units in Group-C (Morinda citrifolia)

**Figure 5:**
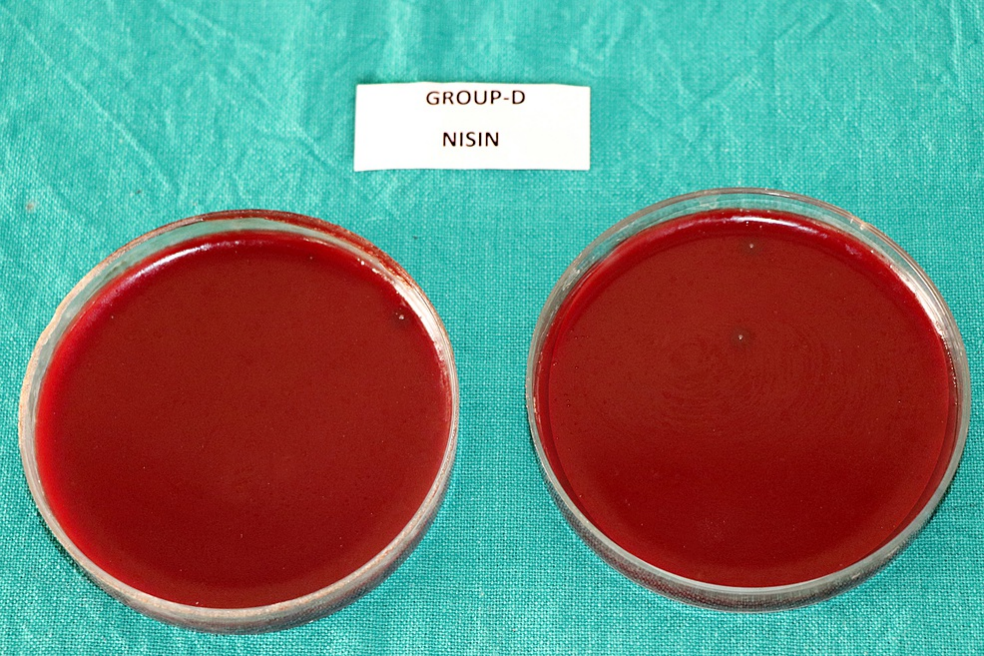
Colony forming units in Group-D (Nisin)

The mean CFU counts of the present study are enumerated in Table 1 and Figure 1. Multiple comparisons among the different groups are displayed in Table 2. The CFU count indicating the number of viable bacterial colonies were found to be highest in Group-A (saline) (Figure 2), followed by Group-C (*M. citrifolia*) (Figure 4). Group-B (2% CHX) (Figure 3) showed the least CFU followed by Group-D (NI) (Figure 5). Multiple comparisons among the different groups, Group-A showed the highest CFU of *E. faecalis* when compared to other experimental groups and the results were statistically significant at p<0.0001. Group-B showed the least CFU of *E. faecalis* when compared to the other experimental groups and the results were statistically significant at p<0.0001. There was no significant difference between Group-B and Group-D with p=0.930. However, the viable CFUs were higher in Group-C when compared to Group-B and Group-D, and the results were statistically significant at p<0.0001.

**Table 1:** Mean comparison between experimental groups (highly significant at p<0.01) CHX: chlorhexidine **p value less than 0.01

	Group A (Saline)	Group B (CHX 2%)	Group C (*Morinda citrifolia*)	Group D (Nisin)
Mean	114.7	0.2	8.00	1.1
Standard deviation	5.85	0.42	3.01	0.99
One way ANOVA value	2808.989			
P value	<0.0001**			

**Table 2:** Intergroup comparison between experimental group (highly significant at p<0.01) CHX: chlorhexidine **p value less than 0.01

Group	Group	P-value
Group A (Saline)	Group B (CHX 2%)	<0.0001**
	Group C (*Morinda citrifolia*)	<0.0001**
	Group D (Nisin)	<0.0001**
Group B (CHX 2%)	Group A (Saline)	<0.0001**
	Group C (*Morinda citrifolia*)	<0.0001**
	Group D (Nisin)	0.930
Group C (*Morinda citrifolia*)	Group A (Saline)	<0.0001**
	Group B (CHX 2%)	<0.0001**
	Group D (Nisin)	<0.0001**
Group D (Nisin)	Group A (Saline)	<0.0001**
	Group B (CHX 2%)	0.930
	Group C (*Morinda citrifolia*)	<0.0001**

## Discussion

The present study compared the antimicrobial efficacy of three medicaments against *E. faecalis*. *E. faecalis* was chosen because of its known role in persistent root canal infection [13]. Human permanent teeth were used as the samples for the present study to correlate with the clinical situation. The CHX group was used to compare with newer intracanal medicaments because it is most commonly used in endodontics [14]. The antimicrobial assessment was done by the direct contact method because it has the advantage of direct and close contact between the test substance and test material, allows gel and paste formulations more homogeneous diffusion conditions than the agar diffusion method [15]. In the present study, on every third day the fresh bacterial sample was inoculated into the roots of the experimental teeth under laminar airflow, for about 21 days in order to prevent medium saturation [10]. For the standardization, all the medicaments were used in a gel form, as the gel form is better retained with longstanding action [1]. Hydroxyethyl cellulose is a non-ionic, highly inert, and water-soluble agent and has been used in various studies for gel formation [16].

According to the present study, 2% CHX reported the highest antibacterial efficacy when compared to that of *M. citrifolia* and studies have shown that CHX gel showed the maximum antimicrobial efficacy against *E. faecalis* [16,17]. 2% CHX demonstrated the highest antibacterial efficacy when compared to that of NI. CHX has good antibacterial efficacy, its potency is due to its ability to alter the cell’s osmotic equilibrium. It has the ability to maintain its antibacterial action for an extended duration owing to its substantivity and optimal microbial activity at a pH of 5.5-7, suggesting it as a root canal irrigation and as an intracanal medicament [18-20]. In the present study, *M. citrifolia* has demonstrated the least CFU of *E. faecalis* when compared to that of the control group, and the mean CFUs were higher than NI and the results are statistically significant at p<0.0001. In the present study, preparation of *M. citrifolia* gel form was used, and studies have shown that 6% concentration of this medicament has effective antimicrobial properties [4].

*M. citrifolia* (Rubiaceae) jiuce (MCJ), commercially known as noni, is indigenous to tropical countries and is considered as a predominant folk medicine. The existence of acubin, L-asperuloside, alizarin, and some other anthraquinone compounds may be responsible for the antibacterial potency of *M. citrifolia* [13]. MCJ has been relatively new to endodontics and has been recommended as an alternative to NaOCl because of its chelating ability to eliminate smear layer and antimicrobial activities, predominantly against anaerobic bacteria such as *E. faecalis* and *Candida albicans* [17]. NI demonstrated the least number of CFU of *E. faecalis* when compared to that of control group, and there was no statistically significant difference (p=0.930) in the mean CFU when NI and 2% CHX were compared, and studies have shown that NI was found to be effective in inhibiting *E. faecalis* in one week and its action was comparable with CHX [18]. NI is a ribosome-synthesized and posttranslationally altered peptide with one lanthionine, four-methyl-lanthionine rings, and unusual residues such as dehydroalanine and dehydrobutyrine [21]. It exerts bactericidal efficacy through pores formation by interacting with a specific molecule “Lipid II” and hinders cell wall synthesis. The chief component of Gram-positive bacterial cell membrane is lipid II. At a nano-molecular level, NI attacks lipid II as a “docking molecule” to form pores on the cell membrane and virtually kills the bacteria [22].

In the present study, gel form of NI was used, studies have shown that 2.5% concentration of this medicament has effective antibacterial properties [18,22]. Several in-vitro studies have determined the efficacy of NI, a bacteriocin, which effectively inhibits *E. faecalis* and *Streptococcus gordonii* cells in solution and within the root canal system. Significantly lower infected dentinal shaving of *E. faecalis* were found with NI when compared to calcium hydroxide [23]. NI has been reported to have anti-biofilm properties and can be used synergistically in combination with conventional therapeutic medicaments [24].

## Conclusions

Within the limitations of the present study, NI was effective in decreasing the bacterial count of *E. faecalis* and its action was found to be comparable with CHX. However, the systemic effect of this medicament, its biocompatibility, allergic potential, and bacterial resistance needs further investigation. Hence, this study can serve as a baseline for future in-vivo studies to analyze the effects of NI and its combinations on eradication of biofilm organisms.
